# Quadrant swapping technique for partial shaded solar photovoltaic system

**DOI:** 10.1038/s41598-025-15120-7

**Published:** 2025-10-01

**Authors:** Hariharasudhan Thangaraj, Bharatiraja Chokkalingam, Sakthivel Aruchamy, Prince Winston David, Sanjeevikumar Padmanaban

**Affiliations:** 1 Department of Electrical and Electronics Engineering, M. Kumarasamy College of Engineering, Karur, Tamilnadu India; 2 Centre for Electric Mobility, Department of Electrical and Electronics Engineering, SRM Institute of Science and Technology, Kattankulathur, Chengalpattu, Tamilnadu-603203 India; 3 Department of Electrical and Electronics Engineering, Sri Ramakrishna Engineering College, Coimbatore, Tamil Nadu 641022 India; 4 Department of Electrical and Electronics Engineering, Kamaraj College of Engineering and Technology, Near Virudhunagar, 625701 India; 5 Department of Electrical engineering, Information Technology and Cybernetics Faculty of Technology, Natural Sciences and Maritime Sciences, University of South-Eastern Norway, Porsgrunn, Norway

**Keywords:** Solar photovoltaic, Partial shading, Reconfiguration techniques, Mismatch loss, Power loss, Output power, Electrical and electronic engineering, Energy harvesting, Renewable energy

## Abstract

Solar energy is one among the most essential renewable sources, and its use is vital to the long-term progress in environmental and energy development. Partial shadowing issues are frequently encountered for solar photovoltaic (PV) systems, and they always influence the PV array’s output power production. There are numerous methods used to reconfigure the PV arrays to extract the possible maximum power. However, those methods have sub-optimal performance in addressing certain possible PV array configurations [series-parallel (SP), total-cross-tie (TCT), SP-TCT, and honeycomb (HC)-TCT]. This method involves the investigation of a 4 × 4 matrix and the incorporation of a switching matrix in the proposed quadrant swapping reconfiguration algorithm. Ten different shading conditions are taken into consideration randomly to confirm the efficacy of this work. A PV array (4 × 4) experimental setup with partial shading yielded an enhanced output power in all the cases. The proposed quadrant swapping method yields the highest output power of 136 W. For three cases, the mismatch and power loss are zero. The proposed quadrant swapping technique improves the output power, and fill factor and reduces the power loss and mismatch loss for all partial shading scenarios. The proposed technique can be applied to large PV systems to enhance their performance. However, they remain profitable on an economic level, as demonstrated by the techno-economic analysis.

## Introduction

The establishment of global power sources depends heavily on renewable energy. The sustainable growth of energy and ecosystems is contingent upon the utilization of solar energy, one among the most significant clean energy sources. Because of its abundance and environmentally beneficial qualities, PV energy is the most significant renewable energy source. Partial shade can be a factor for PV plants, reducing their yield of energy^1–4^. Several methods, including the static and dynamic reconfiguration methods, have been brought out to address partial shading^5,6^. These methods both attempt to increase energy production by minimizing variations in row currents^7,8^.

A new approach that enables the configuration of the module to be dynamically modified, enhancing its performance in a variety of scenarios. Reconfigurability, in contrast to normal PV modules, ensures a more energy output by mitigating challenges like partial shading. 12% more energy was produced by a full-scale prototype of a reconfigurable PV module under partial shadowing than by a static reference module. By efficiently reducing mismatch losses brought on by shading and module faults the suggested reconfiguration approach maximizes power generation under various conditions^9^. The research indicates that the solar PV arrays that are shaded, the study shows that the proposed Chaotic Baker Map (CBM) technique significantly enhances energy output. Comparing the CBM technique to conventional techniques such as the TCT arrangement, test results on array sizes of 4 × 4, 6 × 6, and 8 × 8 showed a reduction in power loss of up to 30%. A 25% improvement in energy extraction was obtained by the CBM techniques under particular shading scenarios, incorporating different shading patterns such as T-shaped, corner, and diagonal shading^10^. The PV array can be dynamically reconfigured for shaded modules in order to increase power output and decrease reverse current owing to the integration of an Insulated Gate Bipolar Transistor (IGBT) model in the proposed design. Nine of the eleven examined cases possessed a consistent decrease in reverse current owing to the modified PV array, showing a notable increase in reliability of 82%. Under several shading environments, the proposed method improves the operating efficiency of PV systems and effectively reduces risks associated with hot spot development^11^. The authors present a newly developed reconfiguration method that uses the Honey Badger Algorithm (HBA) to arrange PV modules in the most effective possible way to maximize energy efficiency if there is partial shade. The HBA-optimized PV array exhibits a power generation improvement of up to 35% compared to conventional approaches, showing the algorithm’s ability to enhance PV system performance^12^.

A modified circuit reconfiguration (MCR) technique for partial shadowing conditions (PSCs) using high-concentration PV modules is presented by Huang *et al*. In comparison to the original series topology, the MCR approach increased power output by about 0.95 W to 4.23 W for square modules and 6.66% to 57.34% for rectangular modules^13^. Aljafari *et al.* proposed a hybrid L-shaped circulating array structure is proposed to maximize the energy output if there are partial shadows. Based on quantitative investigation, mismatch losses are greatly reduced using this approach, and power output improvements are observed between 20% and 30% compared to standard arrangements^14^. The performance of several array topologies, like SP, BL, and TCT, is assessed and compared by Ganesan *et al*. The Bridge-Linked (BL) configuration works substantially better than the others, as shown by numerical data, which show a power gain of up to 25% in shaded conditions^15^. Table 1 revealed the assessment of the existing literature with a detailed description.

**Table 1:** Assessment of the existing literature with a detailed description.

PV size	The number of shading conditions tested	Irradiation level (W/m^2^)	Reconfiguration techniques compared	Maximum power enhancement	Ref.
9×9	4	200, 400, 600, 800, 1000	Total cross-tied, Su-do-Ku, Ancient Chinese Magic Square	382.5 W	^16^
9×9	4	200, 300, 400, 500, 600, 700, 900	Competence Square, Dominance Square, TCT	5630 W	^17^
9×9	5	100, 200, 300, 400, 500, 600, 700, 800, 900	Optimal-Su-do-Ku, TCT, Magic Square, Su-do-Ku	69.3 W	^18^
9×9	4	200, 300, 400, 500, 600, 700, 900	Column index, TCT, Dominance Square, SP	5582 W	^19^
5×5	7	200, 500, 600, 900	SP, Dominance Square, BL, HC, TCT, SP-TCT, BL-TCT, BL-HC	20.62733 W	^20^
6×6	3	100, 200, 300, 400, 500, 600, 700, 1000	SP, HC, TCT, BL, Arrow SudoKu, Skyscraper technique	5566 W	^21^
4×4	7	100, 150, 200, 300, 500, 600, 700, 900, 1000	Dynamic, Couple matching, TCT, Square Dynamic	136 W	^22^
3×3	3	200, 400, 600, 800	SP, HC, TCT, BL, Sudoku, Dragonfly algorithm	57.6 kW	^23^
4×4	10	100, 200, 300, 400, 500, 600, 700, 800, 900, 1000	SP, TCT, SP-TCT, HC-TCT, Proposed method	136 W	Proposed Work

In the specific partial shade conditions and operating parameters assessed in this research, the proposed reconfiguration technique has shown better performance. Conventional topologies like SP, TCT, SP-TCT, and HC-TCT have shown value in reducing mismatch losses, but with more intricate shading patterns, their effectiveness might decrease. On the other hand, given the evaluated conditions, the proposed method delivers a larger energy yield and better power extraction consistency. However, it should be mentioned that these conclusions are based on a comparison with a specific set of current techniques. Recent developments in reconfiguration techniques for photovoltaic (PV) arrays emphasise on maximising the performance in partial shadowing scenarios (PSCs). By contrasting static and dynamic reconfiguration options, the researchers revealed extensive results that indicated that the better dynamic techniques are at reducing shading losses and enhancing energy yield^24^. The researchers presented a sensorless, zero-switch reconfiguration technique for rooftop PV systems that, without the requirement to incorporate additional hardware, showed an average power enhancement of 19.02% above the existing systems^25^. The researchers stated an automatic switching technique that dynamically modifies the connections between PV arrays, enhancing maximum power point tracking (MPPT) by up to 30% as compared to conventional techniques^26^.

**Table:** 

**References**	**Year of Publication**	**Reconfigurations Techniques**	**Intricacy of Control**	**Description of the Hardware**	**Adaptability in Partial Shaded Conditions**	**Efficiency Improvements Reported**
^27^	2024	Examined both static and dynamic approaches	Differs	Differs	Medium to high	Depending on the context
^25^	2023	A comparative analysis of TCT, HC, BL, and SP	Low	Conventional switching	Restricted to static topologies	Moderate
^28^	2023	Zero switch, Sensorless reconfigurations	Low	Minimal	High	An average improvement of 19%
^26^	2023	Sliding Puzzle Pattern	Medium	Re-routing logic is required	High	25% with intricate shading conditions
^29^	2024	GTR PLC	High	Requires hardware	Very high	98% efficiency
Proposed work		Dynamic Hybrid Topology	Medium	Switching matrix	High	30% enhancements

The following concisely summarises the work’s main novelties and contributions:

- A novel quadrant-swapping technique is proposed in this research to enhance the output of the PV array and to enhance the FF by minimizing the ML and PL.
- For the 10 different types of shading patterns, the experiment was conducted in a PV array (4 × 4 matrices).
- The effectiveness of the PV system is quantitatively assessed using output power, power loss, mismatch loss, and fill factor.
- Further, a techno-economic analysis was analyzed to illustrate the potential of the proposed technique.

As compared to the earlier technique of dynamic reconfiguration, the proposed quadrant swapping technique enhanced solar PV systems’ power output. the array’s power output is maximized in the proposed reconfiguration technique using series-parallel connections for a 4 × 4 matrices PV array. PV modules are connected in series to develop a string, followed by the strings being connected in parallel. In industry, it is the most commercially utilized. The proposed quadrant swapping technique increases the fill factor under shading conditions while significantly reducing the ML and PL in terms of performance parameters. The experiment was carried out in a PV array (4 × 4 matrices) for the ten diverse forms of shading patterns for the SP, TCT, SP-TCT, and HC-TCT, and compared with the new proposed quadrant swapping technique. 

The organization of this manuscript is as follows; mathematical modeling is described in the Section"Mathematical modeling". In the Section"Diverse shading scenarios", diverse shading scenarios considered for this work are described. Performance parameters are shown in the Section"Performance parameters". The results and discussions are defined in the Section"Results and discussions". Techno-economic analyses are made in the Section"Techno-economic analysis"followed by the conclusion in the Section"Conclusion".

## Mathematical modeling

**Fig. 1:**
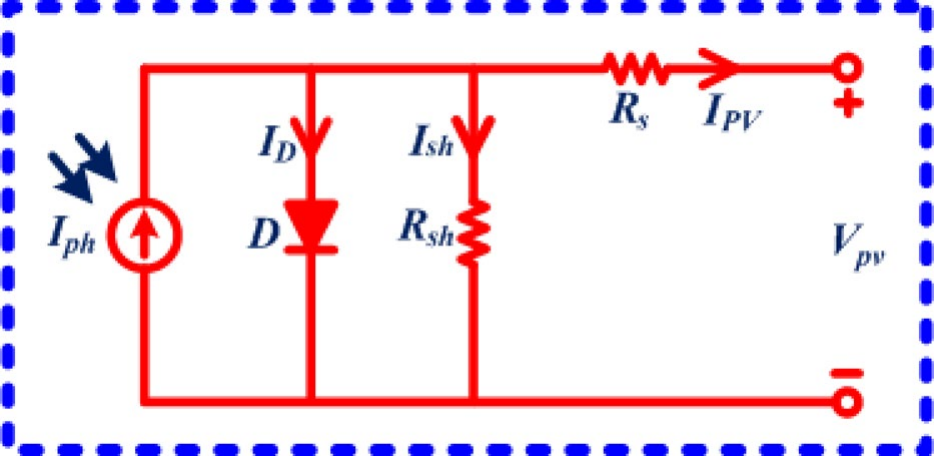
Single diode modelling.

The mathematical representation of the PV cell is one of the aspects influencing the PV system’s effectiveness. The single-diode model is represented in Fig. 1. The PV cell’s single-diode model’s maximum current can be expressed as (1)^29^.,

1 $$\:{I}_{PV}=\:{I}_{ph}-\:{I}_{D}-\:{I}_{sh}$$

where., I_PV_- Photovoltaic current; I_ph_- Photocurrent; I_D_- Diode current; I_sh_- Shunt current.

One can rewrite Eq. (1) in terms of resistance, voltage, and the number of cells in series to get Eq. (2).

2 $$\:{I}_{PV}=\:{I}_{ph}-\:{I}_{D}\left[exp\left\{\frac{{V}_{PV}+{R}_{S}{I}_{PV}}{\frac{nkT}{q}}\right\}-1\right]-\:\left\{\frac{{V}_{PV}+{R}_{S}{I}_{PV}}{{R}_{sh}}\right\}$$

where., V_PV_- The voltage that the PV cell generated; I_PV_- The current that the PV cell generated; R_S_- Series resistance; R_sh_- Shunt resistance; n- No. of cells in series; k- Boltzmann’s constant; T- Temperature; q- Electric charge;

**Table 2:** Module parameters.

S.No.	Module Specifications		
1.	Open circuit voltage	V_oc_	24.8 V
2.	Short circuit current	I_SC_	0.6 A
3.	Maximum voltage	V_m_	20 V
4.	Maximum current	I_m_	0.5 A
5.	Maximum power	P_m_	10 W

*STC- 1000 W/m2; 25℃.

**Fig. 2:**
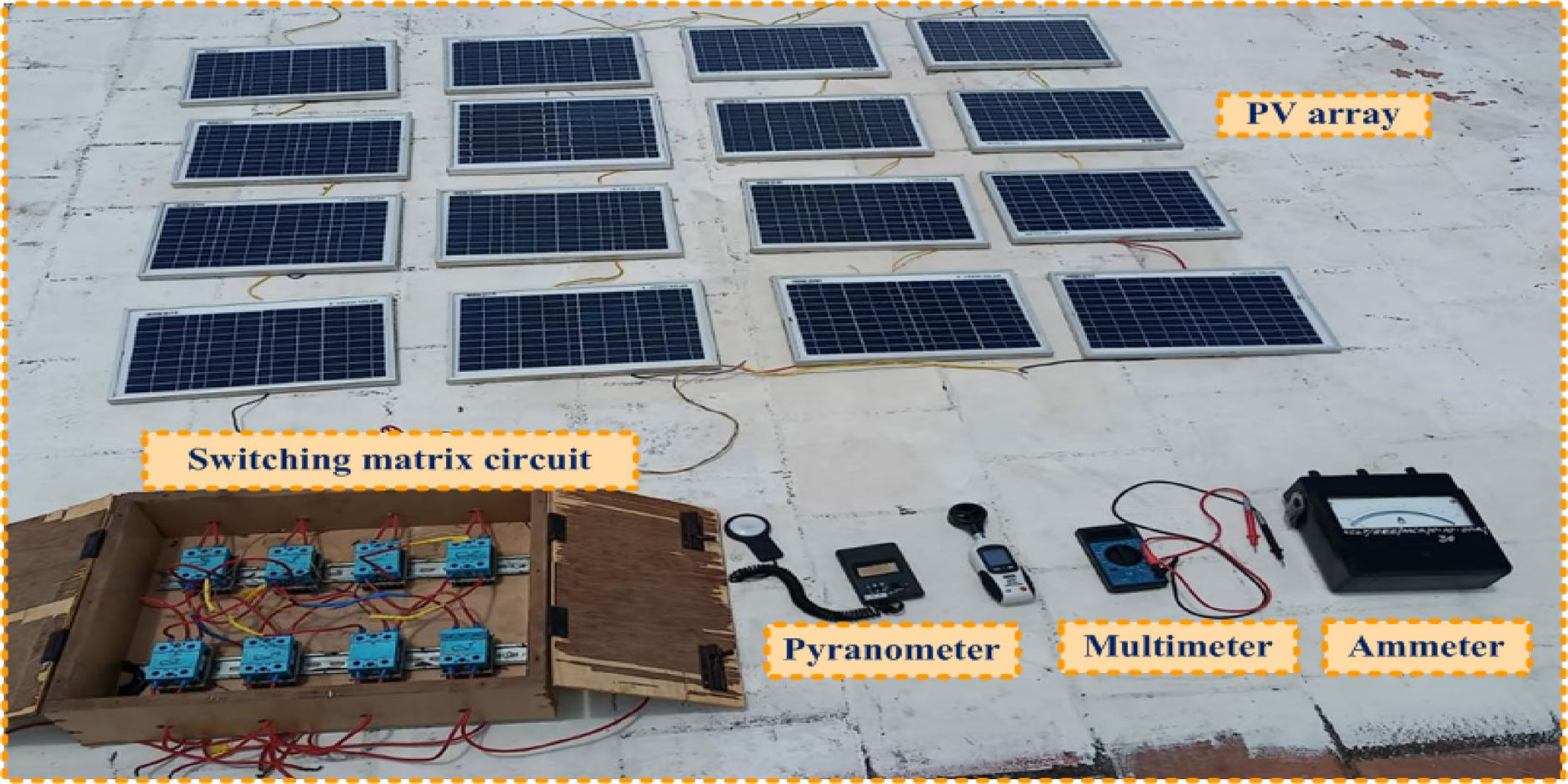
Experimental setup.

**Fig. 3:**
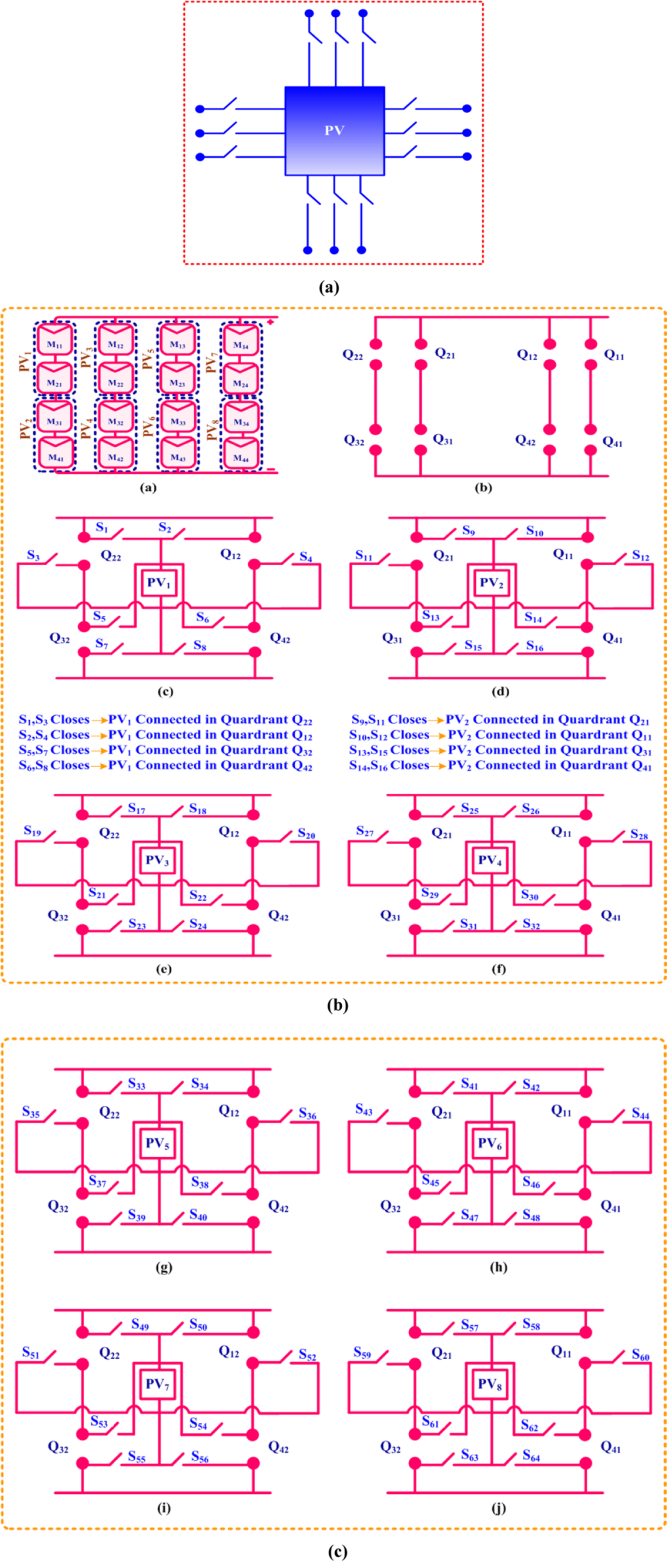
**(a)** Switching circuit. **(b)** Switching circuit of the PV module. **(c)** Switching circuit of the PV module.

The PV module ratings that were considered are represented in Table 2. The experimental setup for the proposed quadrant swapping reconfiguration strategy is shown in Fig. 2. The irradiation on the module is measured with the pyranometer meter. The switching circuit of the proposed quadrant swapping method is illustrated in Fig. 3(a).

### Proposed technique

**Fig. 4:**
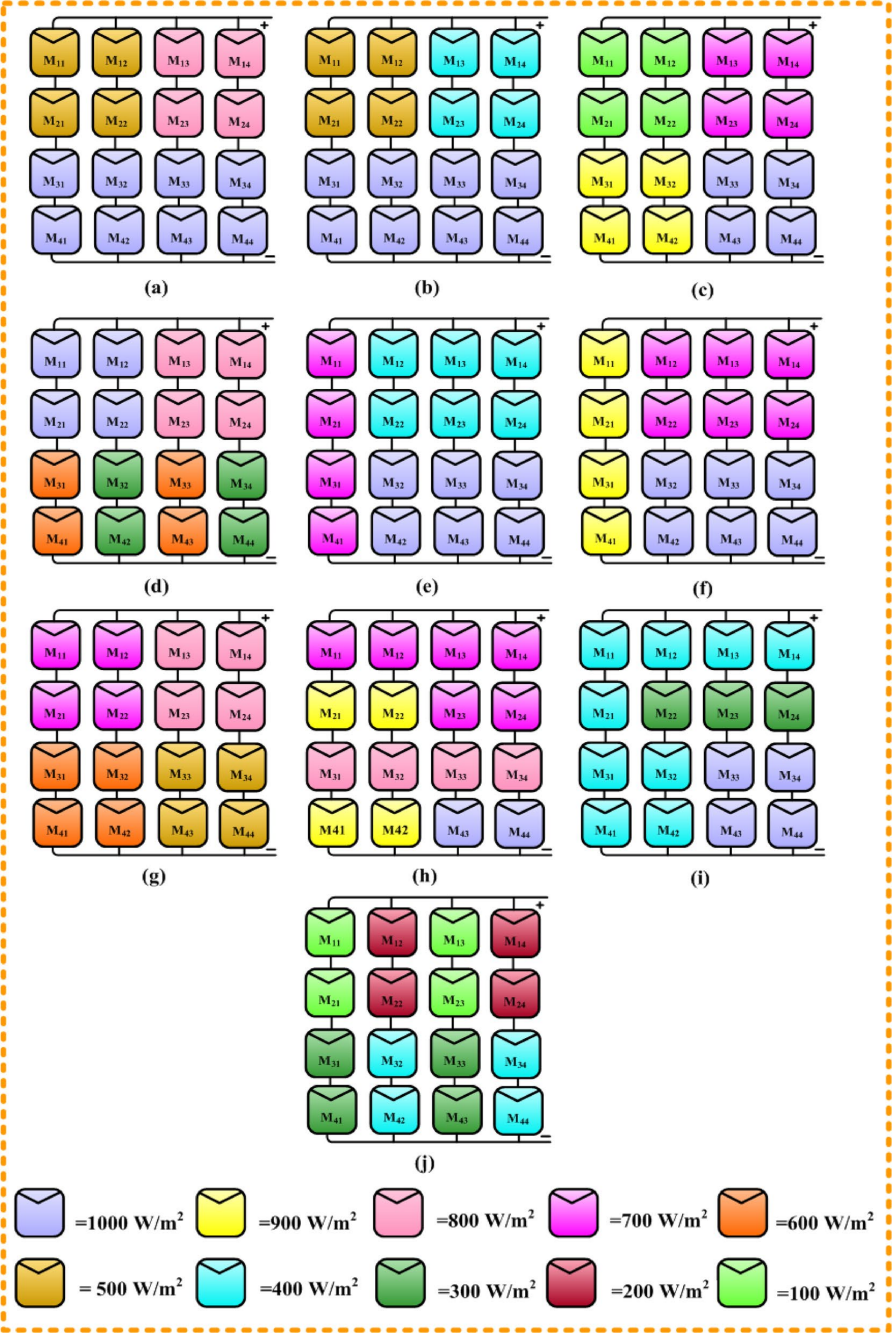
Disperses of different shading for 4 × 4 PV array.

For enhanced power generation, the proposed quadrant swapping technique is employed to the Series-Parallel based electrical wiring of a 4 × 4 matrix photovoltaic array. PV modules (M_11_, M_12_, M_13_, M_14_, M_21_, …, M_44_) are connected in a series-parallel configuration in Fig. 3a. The PV array is segmented into four quadrants: Q_11_ and Q_12_ are quadrant 1, Q_21_ and Q_22_ are quadrant 2, Q_31_ and Q_32_ are quadrant 3, and Q_41_ and Q_42_ are quadrant 4. While shading occurs, the switches (S_1_, S_2_, etc.) regulate the power flow to the various quadrants. PV_1_ is connected to Quadrant Q_22_ in Fig. 3b (c) by closing S_1_ and S_3_. For the other subfigures, a similar logic was applied, and it ensures that the PV module can be allocated to various quadrants as required. To evaluate our proposed quadrant swapping technique under a partial shade environment, the solar PV array is exposed to different levels of irradiation as shown in Fig. 4.

## Diverse shading scenarios

Due to diverse factors, such as surrounding objects like trees, buildings, towers, and clouds, the PV systems can experience uneven irradiation. Diverse irradiation values of certain solar PV modules were used to examine different partial shadowing scenarios. Ten different shading patterns were considered to analyze the proposed quadrant swapping reconfiguration technique. Based on the irradiation values, each panel was assigned a different color. Figure 4 indicates the ten different shading pattern types together with the associated color description.

### Case (a)

The irradiance levels for 4 × 4 matrixes of the case (a) shading pattern are shown in Fig. 4a. In normal irradiation conditions without shade, the PV array (4 × 4) would generate 160 W. A PV array (4 × 4) with ten discrete shading conditions is used to examine the suggested work, as shown in Fig. 4.

- The M_11_, M_12_, M_21_, and M_22_ modules are exposed to 500 W/m^2^ of irradiation.
- The M_13_, M_14_, M_23_, and M_24_ modules are exposed to 800 W/m^2^ of irradiation.
- In addition, 1000 W/m^2^ were delivered to the rest of the PV modules.

### Case (b)

The irradiance levels for 4 × 4 matrixes of the case (b) shading pattern are exposed in Fig. 4b. In normal irradiation conditions without shade, the PV array (4*4) would generate 160 W.

- The M_11_, M_12_, M_21_, and M_22_ modules are exposed to 500 W/m^2^ of irradiation.
- The M_13_, M_14_, M_23_, and M_24_ modules are exposed to 400 W/m^2^ of irradiation. In addition, 1000 W/m^2^ were delivered to the rest of the PV modules.

### Case (c)

The irradiance levels for 4 × 4 matrixes of the case (c) shading pattern are exposed in Fig. 4c.

- The M_11_, M_12_, M_21_, and M_22_ modules are exposed to 100 W/m^2^ of irradiation.
- The M_13_, M_14_, M_23_, and M_24_ modules are exposed to 700 W/m^2^ of irradiation.
- The M_31_, M_32_, M_41_, and M_42_ modules are exposed to 900 W/m^2^ of irradiation. In addition, 1000 W/m^2^ were delivered to the rest of the PV modules.

### Case (d)

The irradiance levels for 4 × 4 matrixes of the case (d) shading pattern are exposed in Fig. 4d.

- The M_13_, M_14_, M_23_, and M_24_ modules are exposed to 800 W/m^2^ of irradiation.
- The M_31_, M_33_, M_41_, and M_43_ and M_32_, M_34_, M_42_, and M_44_ modules are exposed to 600 W/m^2^ and 300 W/m^2^ of irradiation respectively.
- The M_11_, M_12_, M_21_, and M_22_ modules are exposed to 1000 W/m^2^ of irradiation.

### Case (e)

The irradiance levels for 4 × 4 matrixes of the case (e) shading pattern are shown in Fig. 4e.

- The M_11_, M_21_, M_31_, and M_41_ modules are exposed to 700 W/m^2^ of irradiation.
- The M_12_, M_13_, M_14_, and M_22_, M_23_, and M_24_ modules are exposed to 400 W/m^2^ of irradiation. In addition, 1000 W/m^2^ were delivered to the rest of the PV modules.

### Case (f)

The irradiance levels for 4 × 4 matrixes of the case (e) shading pattern are revealed in Fig. 4f.

- The M_11_, M_21_, M_31_, and M_41_ modules are exposed to 900 W/m^2^ of irradiation.
- The M_12_, M_13_, M_14_, and M_22_, M_23_, and M_24_ modules are exposed to 700 W/m^2^ of irradiation. In addition, 1000 W/m^2^ were delivered to the rest of the PV modules.

### Case (g)

The irradiance levels for 4 × 4 matrixes of the case (g) shading pattern are revealed in Fig. 4g.

- The M_11_, M_12_, M_21_, and M_22_ modules are exposed to 700 W/m^2^ of irradiation.
- The M_13_, M_14_, M_23_, and M_24_ modules are exposed to 800 W/m^2^ of irradiation.
- The M_31_, M_32_, M_41_, and M_42_ modules are exposed to 600 W/m^2^ of irradiation.
- The M_33_, M_34_, M_43_, and M_44_ modules are exposed to 500 W/m^2^ of irradiation.

### Case (h)

The irradiance levels for 4 × 4 matrixes of the case (h) shading pattern are revealed in Fig. 4h.

- The M_11_, M_12_, M_13_, M_14_, M_23_, and M_24_ modules are exposed to 700 W/m^2^ of irradiation.
- The M_21_, M_22_, M_41_, and M_42_, modules are exposed to 900 W/m^2^ of irradiation.
- The M_31_, M_32_, M_33_, and M_34_ modules are exposed to 800 W/m^2^ of irradiation. In addition, 1000 W/m^2^ were delivered to the rest of the PV modules.

### Case (i)

The irradiance levels for 4 × 4 matrixes of the case (i) shading pattern are revealed in Fig. 4i.

- The M_22_, M_23_, and M_24_ modules are exposed to 300 W/m^2^ of irradiation.
- The M_33_, M_34_, M_43_, and M_44_ modules are exposed to 1000 W/m^2^ of irradiation. In addition, 1000 W/m^2^ were delivered to the rest of the PV modules.

### Case (j)

The irradiance levels for 4 × 4 matrixes of the case (j) shading pattern are shown in Fig. 4j.

- The M_11_, M_13_, M_21_, and M_23_ modules are exposed to 100 W/m^2^ of irradiation.
- The M_12_, M_14_, M_22_, and M_24_, modules are exposed to 200 W/m^2^ of irradiation.
- The M_31_, M_33_, M_41_, and M_43_ modules are exposed to 300 W/m^2^ of irradiation.
- The M_32_, M_34_, M_42_, and M_44_ modules are exposed to 400 W/m^2^ of irradiation.

**Fig. 5:**
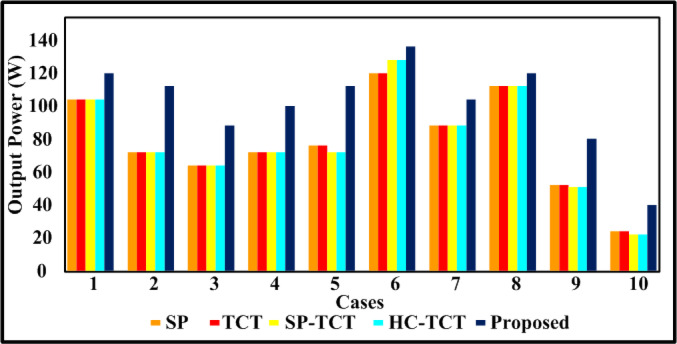
Comparative graph of output power.

Therefore, this paper examines shading patterns implemented in this proposed quadrant swapping reconfiguration methodology’s shaded conditions. Thus, to ensure their maximum power attainment, their performances on each kind of shading condition are analyzed. Figure 3(b), and 3(c) show the execution of the proposed quadrant swapping technique circuit schematic. Through a comparison with the proposed quadrant swapping configuration techniques, the current research effort thoroughly analyses the proposed array develops to validate their effectiveness.

## Performance parameters

The performance parameters of the 4 × 4 PV array are evaluated for the proposed quadrant swapping technique. Output power, mismatch loss, power loss, and fill factor are the evaluation parameters calculated from the proposed quadrant swapping technique evaluated to be compared with the series-parallel, TCT, series-parallel-TCT, and honeycomb-TCT^30,31^.

### Output power

Equation (3) provides the power obtained from the modified solar PV array, which is calculated by multiplying the PV array voltage and current by their respective values.

3 $$\:Output\:power\:\left({P}_{max}\right)=Maximum\:voltage\:\left({V}_{max}\right)\:\times\:Maximum\:current\:\left({I}_{max}\right)$$

where., P_max_-Output power; V_max_-Maximum voltage; I_max_-Maximum current.

### Power loss

The real PV power and its maximum power under partially shaded conditions are compared to get the power loss expressed in an Eq. (4).

4 $$\:Power\:loss\:\left(\%\right)=\:\frac{{P}_{max/STC}-{P}_{real/PSC}}{{P}_{max/STC}}\:\times\:100$$

where., P_max/STC_- Maximum power at standard test condition; P_real/PSC_- Real power at partially shaded condition.

### Mismatch loss

Mismatch loss, which can be computed by Eq. (5), is the variation in power produced by the PV array under STC and the real power generated in PSC.

5 $$\:Mismatch\:loss=\:{P}_{STC}-\:{P}_{PSC}$$

where., P_STC_-Power at standard test condition; P_PSC_-Power at partially shaded condition.

### Fill factor

The effectiveness of a PV module in converting incident sun energy into electrical power is known as the fill factor and is expressed by the Eq. (6).

6 $$\:Fill\:factor=\left[{V}_{max}\times\:{I}_{max}\right]/\:[{V}_{oc}\times\:{I}_{sc}]$$

where., V_max_- Maximum voltage; I_max_- Maximum current; V_OC_- Open circuit voltage; I_SC_- Short circuit current.

## Results and discussions

This study compares existing array configurations from the earliest stages to current advanced methods. The experimental findings of the proposed quadrant-swapping technique for the 10 PSCs are elaborated in this section. The intended objective of the proposed quadrant swapping method is to decrease the mismatch loss and power loss from the PV array under diverse PSCs thereby increasing the output power.

### Case (a)

**Fig. 6:**
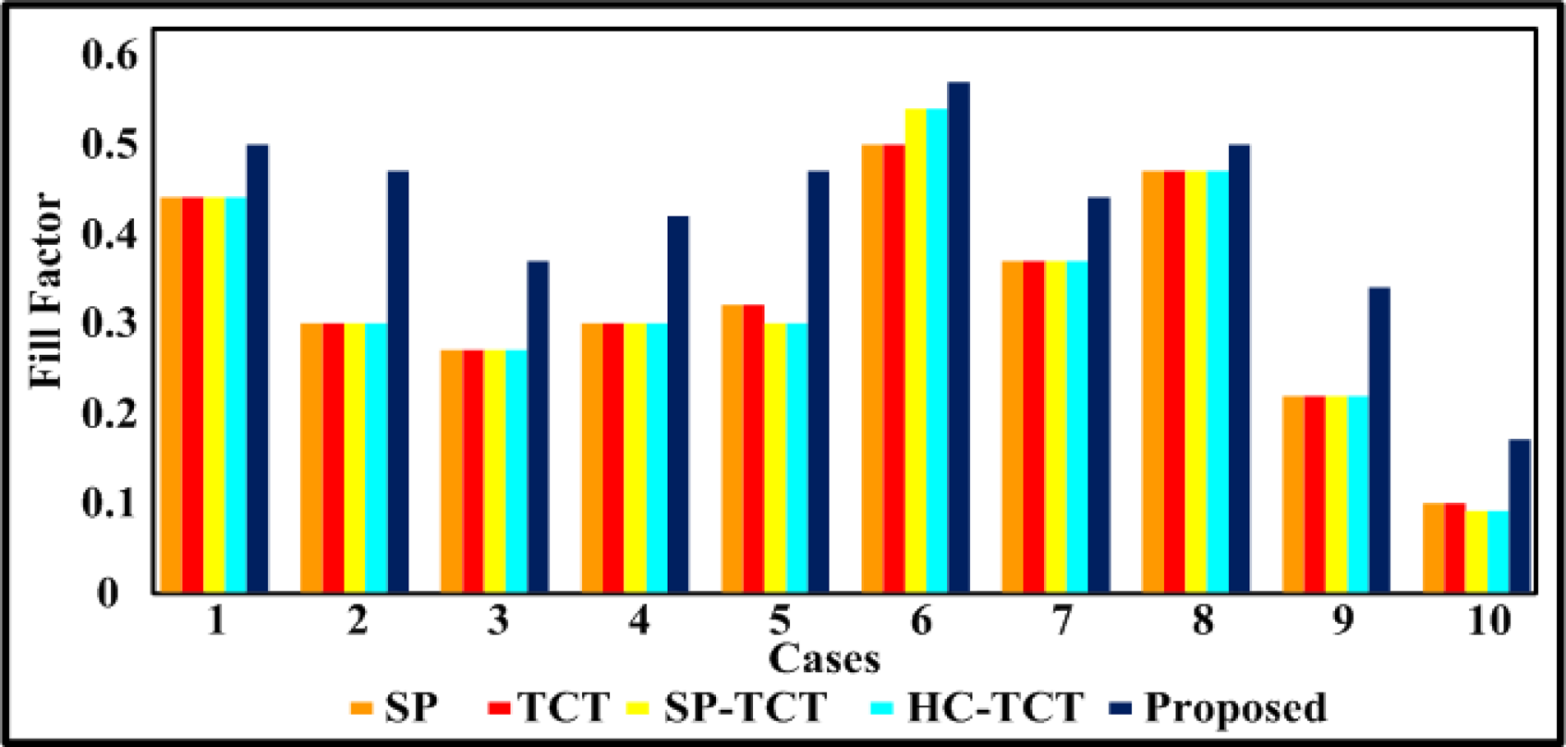
Comparative graph of fill factor.

**Fig. 7:**
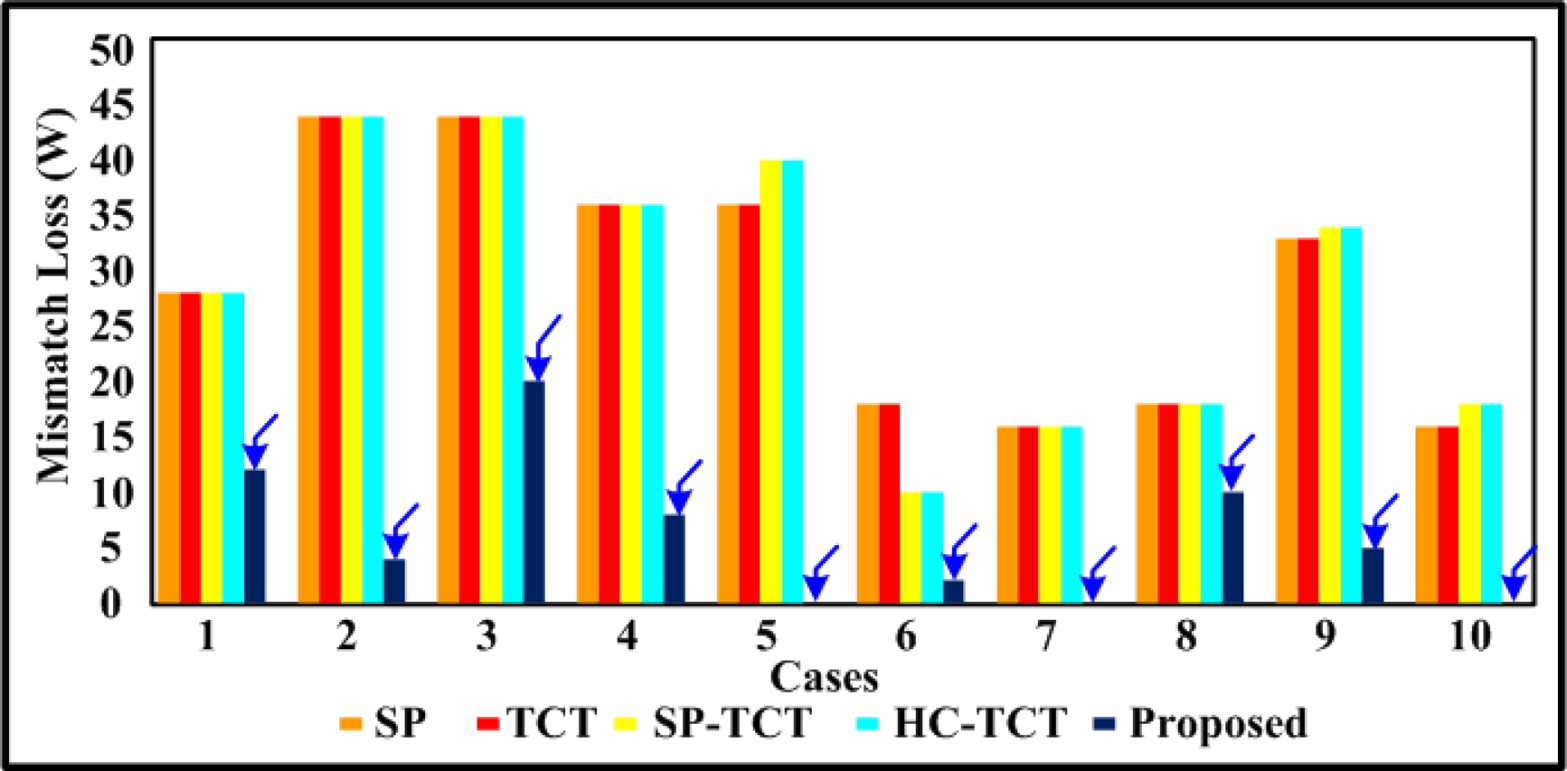
Comparative graph of power loss.

**Fig. 8:**
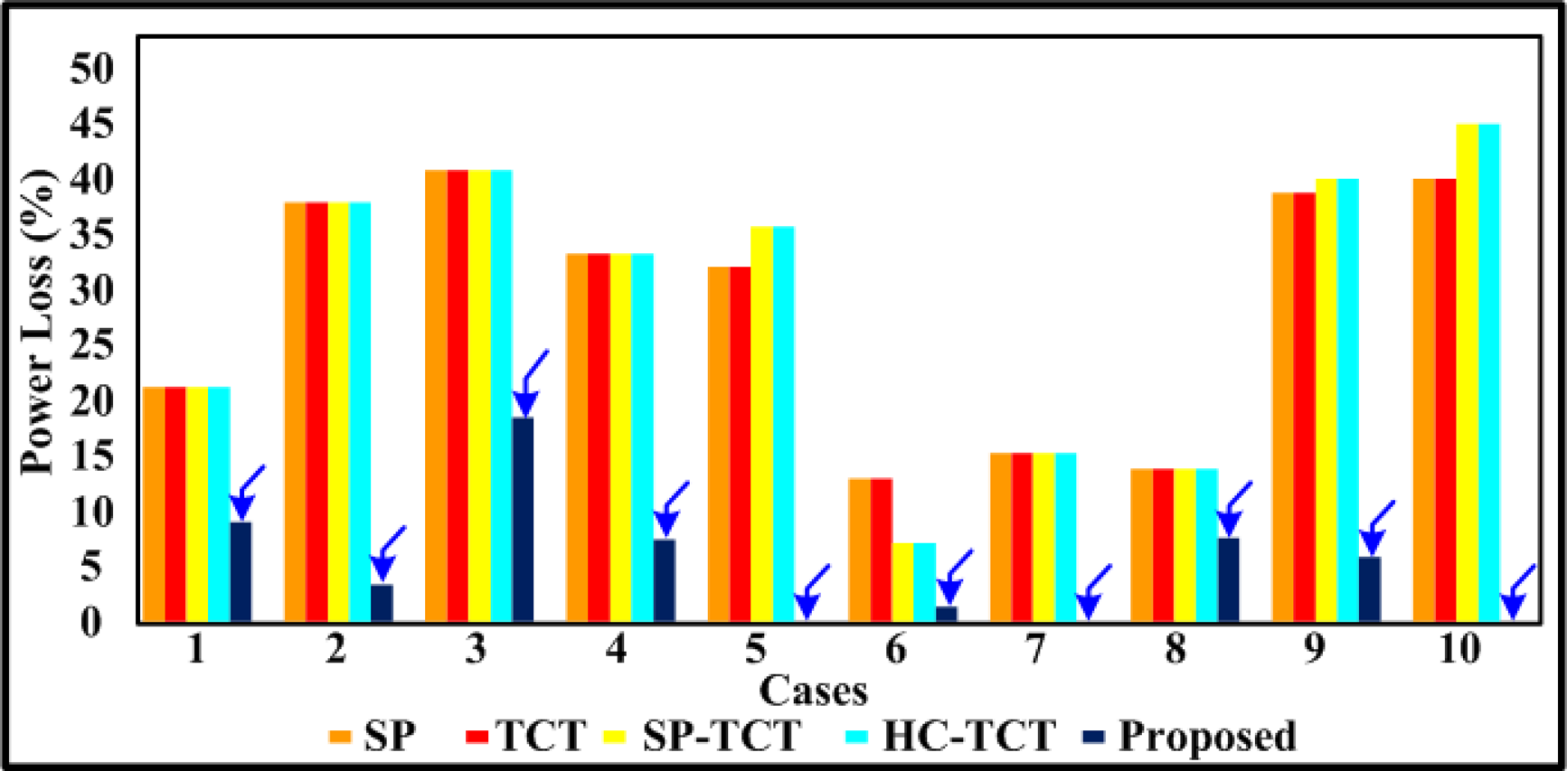
Comparative graph of mismatch loss.

The PV array (4 × 4) is covered by a case (a) shade, which disperses the different irradiations of 1000 W/m^2^, 800 W/m^2^, and 500 W/m^2^. Table 3 includes the results of the assessment parameters that were observed from the case (a). Bar graphs that compare the computed potential variables for case (a) shading make the comparative results easy to see in Figs. 5, 6, 7 and 8. Figure 8 shows that the proposed quadrant swapping technique has a PL of 9% and which is less compared to other configuration techniques. Figure 6 reveals that the proposed quadrant swapping technique achieves an FF of 0.5 for case (a). With the proposed quadrant swapping configuration technique, maximum power is generated with less mismatch loss than conventional arrays.

**Table 3:** Results of the assessment of parameters’ investigation.

Techniques	I (A)	V (V)	*P* (W)	ML (W)	PL (%)	FF
Case (a)						
SP	1.3	80	104	28	21	0.44
TCT	1.3	80	104	28	21	0.44
SP-TCT	1.3	80	104	28	21	0.44
HC-TCT	1.3	80	104	28	21	0.44
Proposed	**1.5**	**80**	**120**	**12**	**9**	**0.5**
Case (b)						
SP	0.9	80	72	44	38	0.3
TCT	0.9	80	72	44	38	0.3
SP-TCT	0.9	80	72	44	38	0.3
HC-TCT	0.9	80	72	44	38	0.3
Proposed	**1.4**	**80**	**112**	**4**	**3.4**	**0.47**
Case (c)						
SP	0.8	80	64	44	40.7	0.27
TCT	0.8	80	64	44	40.7	0.27
SP-TCT	0.8	80	64	44	40.7	0.27
HC-TCT	0.8	80	64	44	40.7	0.27
Proposed	**1.1**	**80**	**88**	**20**	**18.5**	**0.37**
Case (d)						
SP	0.9	80	72	36	33.3	0.3
TCT	0.9	80	72	36	33.3	0.3
SP-TCT	0.9	80	72	36	33.3	0.3
HC-TCT	0.9	80	72	36	33.3	0.3
Proposed	**1.25**	**80**	**100**	**8**	**7.4**	**0.42**
Case (e)						
SP	0.95	80	76	36	32.1	0.32
TCT	0.95	80	76	36	32.1	0.32
SP-TCT	0.9	80	72	40	35.7	0.3
HC-TCT	0.9	80	72	40	35.7	0.3
Proposed	**1.4**	**80**	**112**	**0**	**0**	**0.47**
Case (f)						
SP	1.5	80	120	18	13	0.5
TCT	1.5	80	120	18	13	0.5
SP-TCT	1.6	80	128	10	7.2	0.54
HC-TCT	1.6	80	128	10	7.2	0.54
Proposed	**1.7**	**80**	**136**	**2**	**1.4**	**0.57**
Case (g)						
SP	1.1	80	88	16	15.3	0.37
TCT	1.1	80	88	16	15.3	0.37
SP-TCT	1.1	80	88	16	15.3	0.37
HC-TCT	1.1	80	88	16	15.3	0.37
Proposed	**1.3**	**80**	**104**	**0**	**0**	**0.44**
Case (h)						
SP	1.4	80	112	18	13.8	0.47
TCT	1.4	80	112	18	13.8	0.47
SP-TCT	1.4	80	112	18	13.8	0.47
HC-TCT	1.4	80	112	18	13.8	0.47
Proposed	**1.5**	**80**	**120**	**10**	**7.6**	**0.5**
Case (i)						
SP	0.65	80	52	33	38.8	0.22
TCT	0.65	80	52	33	38.8	0.22
SP-TCT	0.64	80	51	34	40	0.22
HC-TCT	0.64	80	51	34	40	0.22
Proposed	**1**	**80**	**80**	**5**	**5.88**	**0.34**
Case (j)						
SP	0.3	80	24	16	40	0.1
TCT	0.3	80	24	16	40	0.1
SP-TCT	0.28	80	22	18	45	0.09
HC-TCT	0.28	80	22	18	45	0.09
Proposed	**0.5**	**80**	**40**	**0**	**0**	**0.17**

### Case (b)

The PV array (4 × 4) is covered by a case (b) shade, which disperses the different irradiations of 1000 W/m^2^, 500 W/m^2^, and 400 W/m^2^. Table 3 includes the results of the assessment parameters that were observed from case (b). Bar graphs that compare the computed potential variables for case (b) shading make the comparative results easy to see in Figs. 5, 6, 7 and 8. Figure 8 shows that the proposed quadrant swapping technique has a PL of 3.4% and which is less compared to other configuration techniques. Figure 6 reveals that the proposed quadrant swapping technique achieves an FF of 0.47 for case (b). With the proposed quadrant swapping configuration technique, maximum power is generated with less mismatch loss than conventional arrays.

### Case (c)

The PV array (4 × 4) is covered by a case (c) shade, which disperses the different irradiations of 1000 W/m^2^, 900 W/m^2^, 700 W/m^2^, and 100 W/m^2^. As seen in Fig. 5, the proposed quadrant swapping technique has produced 88 W of output power for case (c). Table 3 includes the results of the assessment parameters that were observed from case (c). Figure 8 shows that the proposed quadrant swapping technique has a PL of 18.5% which is less compared to other configuration techniques. With the proposed quadrant swapping configuration technique, maximum power is generated with less mismatch loss than conventional arrays. Figure 6 reveals that the proposed technique achieves an FF of 0.37 for case (c).

### Case (d)

The PV array (4 × 4) is covered by a case (d) shade, which disperses the different irradiations of 1000 W/m^2^, 800 W/m^2^, 600 W/m^2^, and 300 W/m^2^. As seen in Fig. 5, the proposed quadrant swapping technique has produced 100 W of output power for case (d). Table 3 includes the results of the assessment parameters that were observed from case (d). Figure 8 shows that the proposed quadrant swapping technique has a PL of 7.4% which is less compared to other configuration techniques. Based on shade distribution and power generation, this proposed quadrant-swapping method outperforms than conventional arrays. Figure 6 reveals that the proposed technique achieves an FF of 0.42 for case (d).

### Case (e)

The PV array (4 × 4) is covered by a case (e) shade, which disperses the different irradiations of 1000 W/m^2^, 700 W/m^2^, and 400 W/m^2^. As seen in Fig. 5, the proposed quadrant swapping technique has produced 112 W of output power for case (e). Table 3 includes the results of the assessment parameters that were observed from case (e). Figures 7 and 8 show that the proposed quadrant swapping technique has an ML and PL of 0%. Figure 6 reveals that the proposed technique achieves an FF of 0.47 for case (e). With the proposed quadrant swapping configuration technique, maximum power is generated over conventional arrays. This case (e) has zero mismatch losses.

### Case (f)

The PV array (4 × 4) is covered by a case (f) shade, which disperses the different irradiations of 1000 W/m^2^, 900 W/m^2^, and 700 W/m^2^. As seen in Fig. 5, the proposed quadrant swapping technique has produced 136 W of output power for case (f). Table 3 includes the results of the assessment parameters that were observed from case (f). Figures 7 and 8 show that the proposed quadrant swapping technique has an ML and PL of 2 W and 1.4% respectively. Figure 6 reveals that the proposed quadrant swapping technique achieves an FF of 0.57 for case (f). With the proposed quadrant swapping configuration technique, maximum power is generated with less mismatch loss than conventional arrays.

### Case (g)

The PV array (4 × 4) is covered by a case (g) shade, which disperses the different irradiations of 800 W/m^2^, 700 W/m^2^, 600 W/m^2^, and 500 W/m^2^. As seen in Fig. 5, the proposed quadrant swapping technique has produced 104 W of output power for case (g). Table 3 includes the results of the assessment parameters that were observed from case (g). Figure 7 shows that the proposed quadrant swapping technique has an ML of 0 W. Figure 6 reveals that the proposed technique achieves an FF of 0.44 for case (g). With the proposed quadrant swapping configuration technique, maximum power is generated over conventional arrays. This case (g) has zero mismatch losses.

**Fig. 9:**
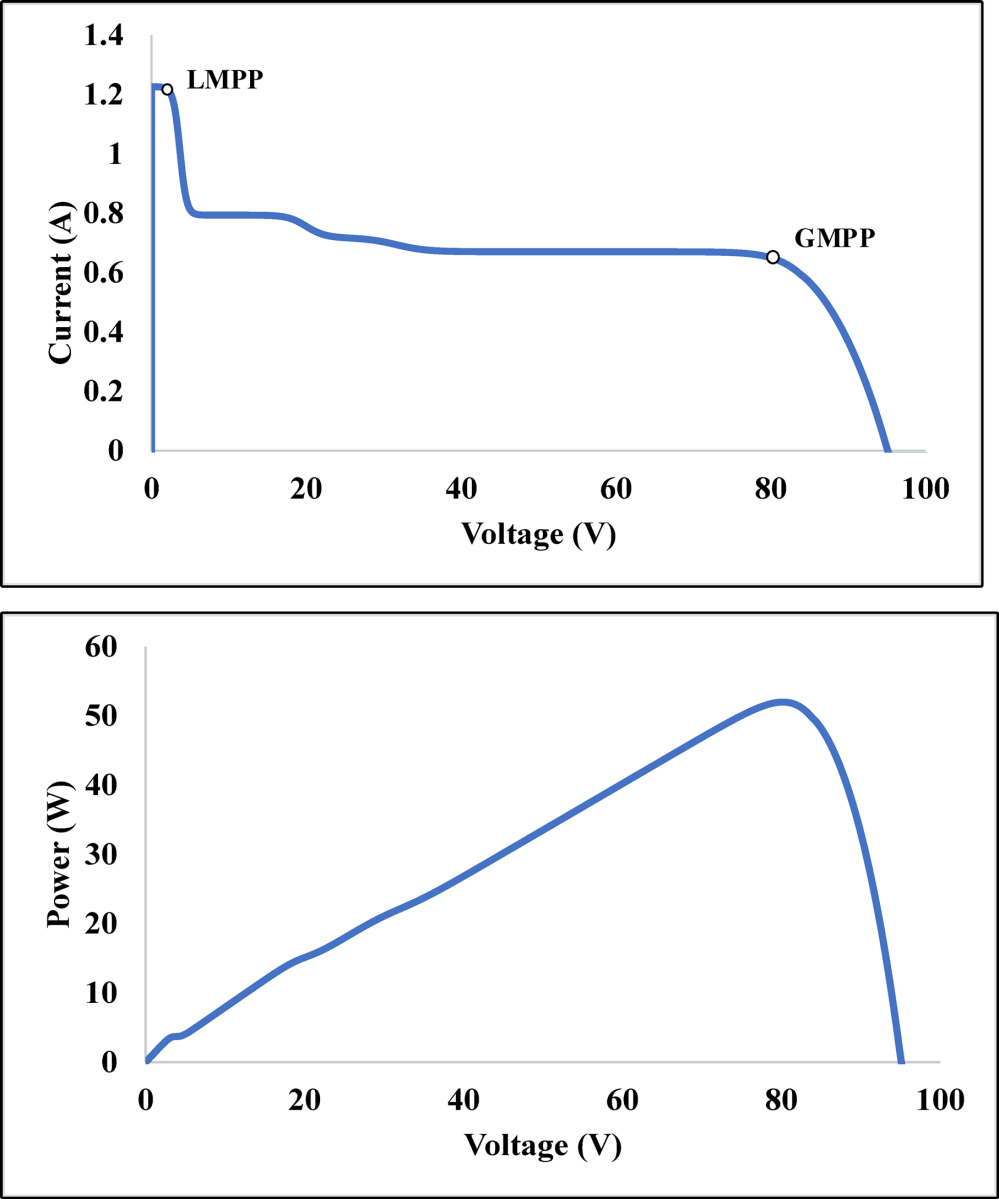
(**a**) V-I characteristics of partially shaded PV panel, (**b**) P-V characteristics of partially shaded PV panel.

### Case (h)

The PV array (4 × 4) is covered by a case (h) shade, which disperses the different irradiations of 1000 W/m^2^, 900 W/m^2^, 800 W/m^2,^ and 700 W/m^2^. As seen in Fig. 5, the proposed quadrant swapping technique has produced 120 W of output power for case (h). Table 3 includes the results of the assessment parameters that were observed from case (h). Figures 7 and 8 show that the proposed quadrant swapping technique has an ML and PL of 10 W and 7.6% respectively. Figure 6 reveals that the proposed quadrant swapping technique achieves an FF of 0.5 for case (h). With the proposed quadrant swapping configuration technique, maximum power is generated with less mismatch loss than conventional arrays.

### Case (i)

The PV array (4 × 4) is covered by a case (i) shade, which disperses the different irradiations of 1000 W/m^2^, 400 W/m^2^, and 300 W/m^2^. As seen in Fig. 5, the proposed quadrant swapping technique has produced 80 W of output power for case (i). Table 3 includes the results of the assessment parameters that were observed from case (i). Figures 7 and 8 shows that the proposed quadrant swapping technique has an ML and PL of 5 W and 5.88% respectively. Figure 6 reveals that the proposed quadrant swapping technique achieves an FF of 0.34 for case (i) and for other configurations it is 0.22. Based on shade distribution and power generation, this proposed quadrant-swapping method outperforms than conventional arrays.

### Case (j)

The PV array (4 × 4) is covered by a case (j) shade, which disperses the different irradiations of 400 W/m^2^, 300 W/m^2^, 200 W/m^2^, and 100 W/m^2^. As seen in Fig. 5, the proposed quadrant swapping technique has produced 40 W of output power for case (j). Table 3 includes the results of the assessment parameters that were observed from case (j). Figures 7 and 8 show that the proposed quadrant swapping technique has an ML and PL of 0 W and 0% respectively. Figure 6 reveals that the proposed quadrant swapping technique achieves an FF of 0.17 for case (j). With the proposed quadrant swapping configuration technique, maximum power is generated over conventional arrays. This case (j) has zero mismatch losses.

For case (f), the maximum output power of 136 W is achieved due to the nature of the shading scenarios. For cases (e), (g), and (j), the mismatch and power loss are zero due to the nature of the PSCs. The proposed method outperforms to square-based PSCs. In distributed and cumulative square-based PSCs, our suggested approach performs effectively. The techno-economic analysis is carried out on the commercial model and shown in the section below. The V-I and P-V curve for the partially shaded PV module is depicted in Fig. 9 of case 9.

**Fig. 10:**
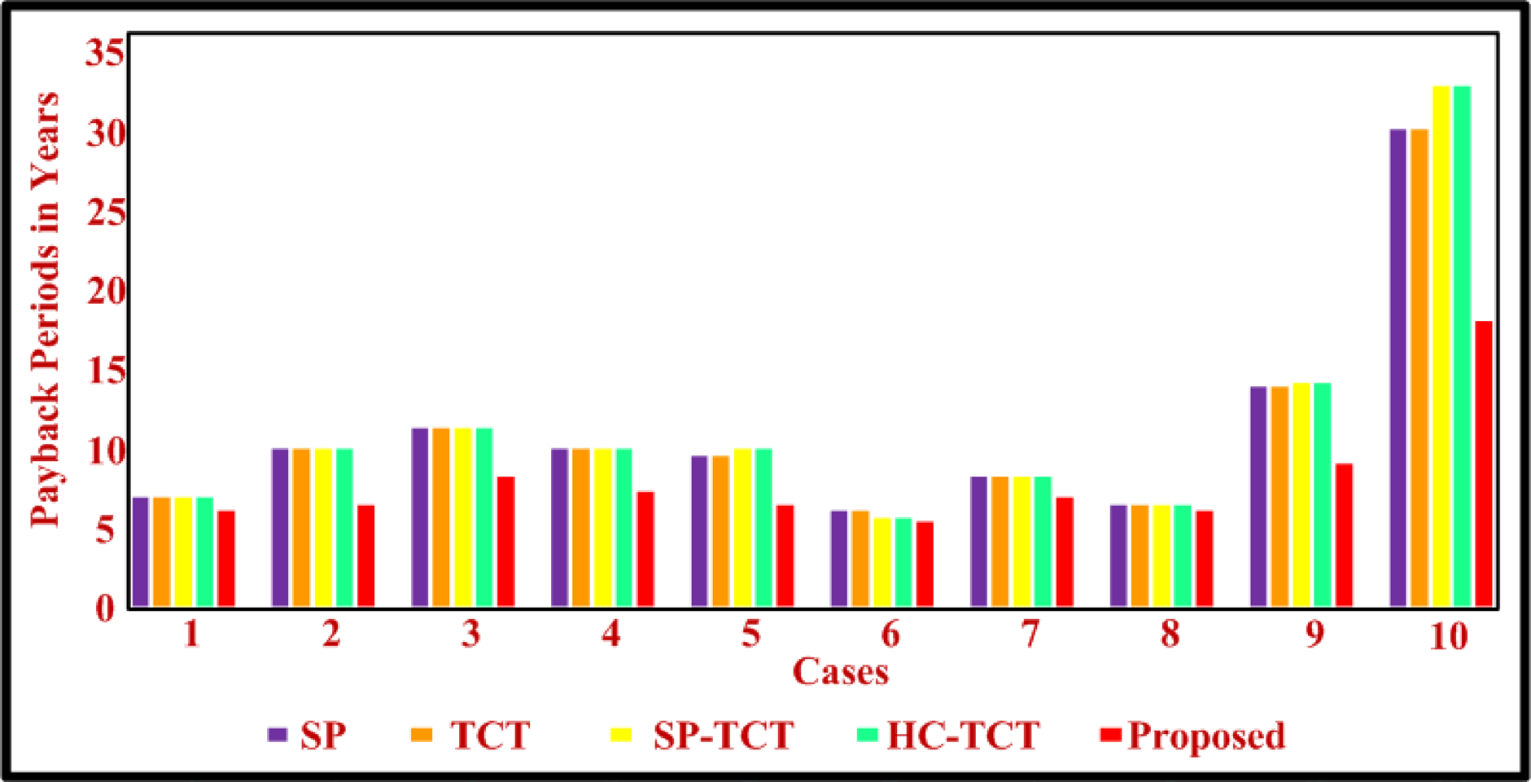
Comparison of payback period.

## Techno-economic analysis

One of the main factors influencing a module’s economic significance is its cost. Rodriguez-Gallegos et al. evaluated the Levelized Cost of Electricity (LCOE) of the PV system^30–32^. Table 4 revealed the cost analysis of the PV system under PSCs.

**Table 4:** Shows the expected cost analysis of the PV system unde PSCs.

Cases	Case- (a)	Case- (b)	Case- (c)	Case- (d)	Case- (e)	Case- (f)	Case- (g)	Case- (h)	Case- (i)	Case- (j)
Cost of the module in Rs	12,299	12,299	12,299	12,299	12,299	12,299	12,299	12,299	12,299	12,299
Energy cost per kWh in Rs.	8.5	8.5	8.5	8.5	8.5	8.5	8.5	8.5	8.5	8.5
Plant load factor	0.2	0.2	0.2	0.2	0.2	0.2	0.2	0.2	0.2	0.2
Peak power in kW	0.12	0.112	0.088	0.1	0.112	0.136	0.104	0.12	0.08	0.04
Daily energy generated by the module in kWh	0.66	0.616	0.484	0.55	0.616	0.748	0.572	0.66	0.44	0.22
Daily energy generated cost in Rs.	5.61	5.236	4.114	4.675	5.236	6.358	4.862	5.61	3.74	1.87
Energy generated per annum cost in Rs.	2047.7	1911.14	1501.61	1706.38	1911.14	2320.67	1774.63	2047.65	1365.1	682.55
Payback period in years	6.006	6.4354	8.1905	7.2076	6.4354	5.299	6.930	6.006	9.009	18.01

The estimated payback period for the module under case (a) is 6 years, moreover, it has been 7 years for SP, TCT, SP-TCT, and HC-TCT. A comparison graph of the payback period for all the cases is shown in Fig. 10. In contrast, the module that uses a proposed technique has a lifespan of 6 years, which is 13.33% higher than the module that uses a conventional SP for case (a).

## Conclusion

A Quadrant Swapping Technique has been successfully demonstrated to mitigate power loss in PV arrays and mismatch losses in PV arrays that are subjected to partial shading. Through experimental validation across ten various shading conditions, the approach consistently delivered better performance compared to typical configurations such as SP, TCT, SP-TCT, and HC-TCT. The overall power output, fill factor were all effectively enhanced by the proposed quadrant swapping technique. However, scenario (f) had the highest output power of 136 W, a corresponding FF of 0.57, ML of 2 W, and PL of 1.4%. Conversely, the lowest measured output power was 80 W, with a FF of 0.34, ML of 5 W, and PL of 5.88%. However, from a techno-economic analysis, the lifespan of the suggested method is 6 years, 13.33% longer than that of the module that employs a traditional SP in scenario (a). The findings show that, in comparison with the other configurations (SP, TCT, SP-TCT, and HC-TCT), the proposed quadrant swapping reconfiguration technique is deemed worthy and achieves superior outcomes. This novel technique is applied to large solar power plants to achieve improved output power with less mismatch loss. Further switch reduction may enhance solar PV power output in the future, and grid-connected systems’ array reconfiguration techniques provide an incredible amount of PV system research approaches.

## Data Availability

The datasets used and/or analyzed during the current study are available from the corresponding author on reasonable request.
